# A glimpse into viral warfare: decoding the intriguing role of highly pathogenic coronavirus proteins in apoptosis regulation

**DOI:** 10.1186/s12929-024-01062-1

**Published:** 2024-07-13

**Authors:** Leyi Cheng, Yajuan Rui, Yanpu Wang, Shiqi Chen, Jiaming Su, Xiao-Fang Yu

**Affiliations:** 1 Cancer Institute (Key Laboratory of Cancer Prevention and Intervention, China National Ministry of Education), The Second Affiliated Hospital, Zhejiang University School of Medicine, Zhejiang University, Hangzhou, Zhejiang 310009 China; 2 Cancer Center, Zhejiang University, Hangzhou, Zhejiang 310058 China

**Keywords:** Apoptosis, Highly Pathogenic Coronavirus, Antiviral Drugs

## Abstract

Coronaviruses employ various strategies for survival, among which the activation of endogenous or exogenous apoptosis stands out, with viral proteins playing a pivotal role. Notably, highly pathogenic coronaviruses such as SARS-CoV-2, SARS-CoV, and MERS-CoV exhibit a greater array of non-structural proteins compared to low-pathogenic strains, facilitating their ability to induce apoptosis via multiple pathways. Moreover, these viral proteins are adept at dampening host immune responses, thereby bolstering viral replication and persistence. This review delves into the intricate interplay between highly pathogenic coronaviruses and apoptosis, systematically elucidating the molecular mechanisms underpinning apoptosis induction by viral proteins. Furthermore, it explores the potential therapeutic avenues stemming from apoptosis inhibition as antiviral agents and the utilization of apoptosis-inducing viral proteins as therapeutic modalities. These insights not only shed light on viral pathogenesis but also offer novel perspectives for cancer therapy.

## Highlights

• Apoptosis plays an important role in the pathogenesis of the highly pathogenic coronavirus

• The structural and non-structural proteins of highly pathogenic coronaviruses exert significant influence over apoptosis regulation

• Apoptosis inhibitors exhibits promising antiviral effects, thereby presenting a potential avenue for the development of novel therapeutics targeting COVID-19.

## Background

**Fig. 1:**
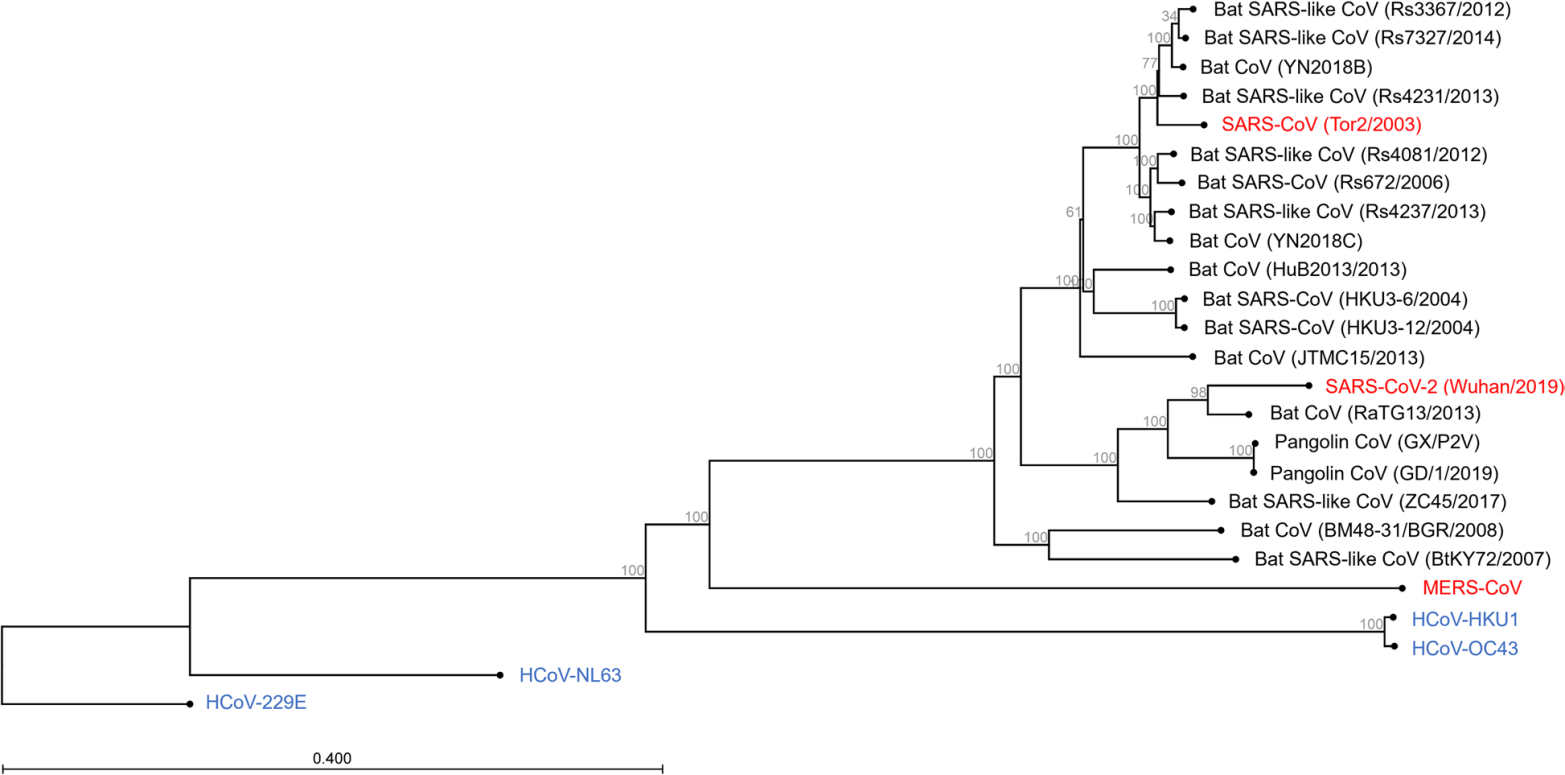
Phylogenetic tree of highly pathogenic coronavirus (red), low-pathogenic coronavirus (blue) and the other SARS-related coronavirus. Reference sequences of representative coronaviruses include phylogenetic analysis was performed with the CLC program by the neighbor-joining method on the basis of the Kimura two-parameter model

Coronaviruses are a large family of viruses that cause illness in both animals and humans. They are divided into four genera: α, β, γ and δ. Among these, humans are primarily susceptible to coronaviruses from the α and β genera. The β genera, in particular, include three highly pathogenic species: severe acute respiratory syndrome coronavirus 2 (SARS-CoV-2), responsible for the Corona Virus Disease 2019 (COVID-19) pandemic, as well as severe acute respiratory syndrome coronavirus (SARS-CoV) and middle east respiratory syndrome coronavirus (MERS-CoV) (Fig. 1). On the other hand, there are four low-pathogenic species commonly associated with mild respiratory symptoms, namely human coronavirus (HCoV) 229E, NL63, OC43, and HKU1 (Fig. 1), which are classified under the α and β genera. Bat SARS-like coronaviruses belong to β genera also show potential for human emergence [1, 2] (Fig. 1). Infection with these low-pathogenic coronaviruses typically results in symptoms resembling the common cold [3].

COVID-19, caused by SARS-CoV-2, emerged in late 2019 and has become a global pandemic. By the end of 2023, there have been over 770 million reported cases and over 6.9 million deaths worldwide [4]. Previous large-scale coronavirus outbreaks include SARS in 2002 (caused by SARS-CoV) and MERS in 2012 (caused by MERS-CoV). The cumulative number of infections for SARS and MERS was a approximately 8,000 [5] and 2,600 [6], respectively, with cumulative death toll of 774 [5] and 936 [6].

**Fig. 2:**
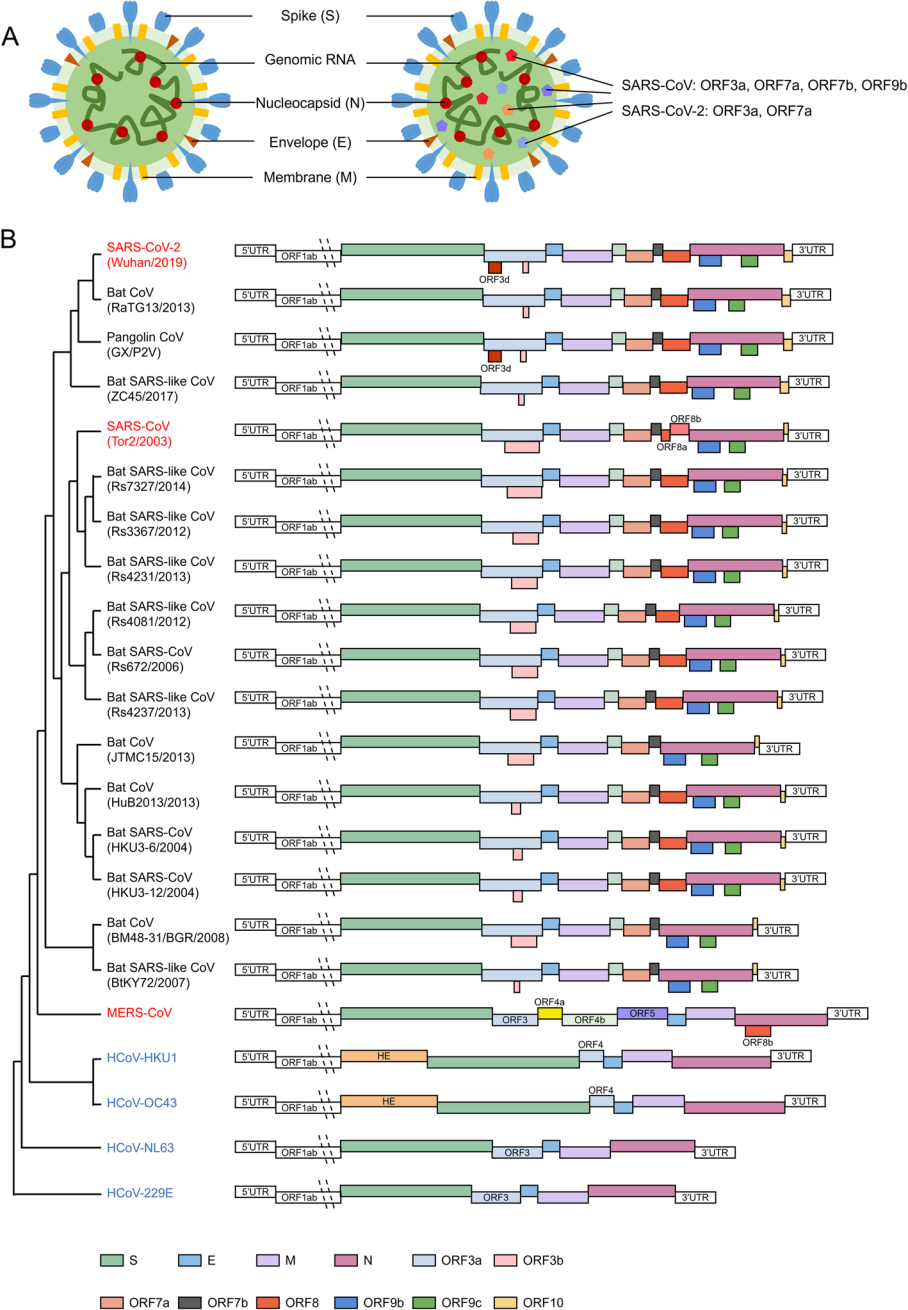
General structural pattern diagram and genome of coronavirus. **A**, Coronavirus particles include E, M, N, S, genomic RNA and secondary components such as ORF3a, ORF7a, ORF7b, ORF9b of SARS-CoV and ORF3a, ORF7a of SARS-CoV-2. **B**, Schematic diagram of the genomic organization and encoded proteins of SARS-related coronavirus. The highly pathogenic coronaviruses (red) encode more non-structural proteins

The virus particles of SARS-CoV, MERS-CoV, and SARS-CoV-2 include genomic RNA and four structural proteins, spike (S), envelope (E), membrane (M) and nucleocapsid (N). Non-structural proteins are not necessarily incorporated into the virus particles, except ORF3a, ORF7a, ORF7b, ORF9b of SARS-CoV [7, 8] and ORF3a, ORF7a of SARS-CoV-2 [9] (Fig. 2A). SARS-CoV, MERS-CoV, and SARS-CoV-2 are positive-sense, single-stranded RNA viruses with genomes of about 30,000 bases in length. Their genomes include 5' end cap-like structure, structural proteins S, E, M and N, non-structural proteins, and 3'-end poly A tails [10–15] (Fig. 2B). Comparing different human-susceptible coronavirus genomes, it’s evident that highly pathogenic coronaviruses encode more non-structural proteins than low-pathogenic coronaviruses (Fig. 2B). Many studies have demonstrated that different non-structural proteins can help highly pathogenic coronaviruses evade host immune responses more effectively and promote viral replication in different ways. Regulation of apoptosis is one of the important way [16–25]. In this review, we summarize the current knowledge of the apoptosis induced by highly pathogenic coronaviruses and their molecular mechanisms, as well as the potential applications of apoptosis inhibitors as antiviral drugs.

### Apoptosis signal transduction

Apoptosis, a programmed cell death process, was originally proposed by J.F. Kerr in 1972 [26]. The classical apoptosis is primarily categorized into three pathways based on the origin of the apoptotic signal: the endogenous endoplasmic reticulum (ER) stress pathway, the endogenous DNA damage pathway and the exogenous death receptor pathway.

**Fig. 3:**
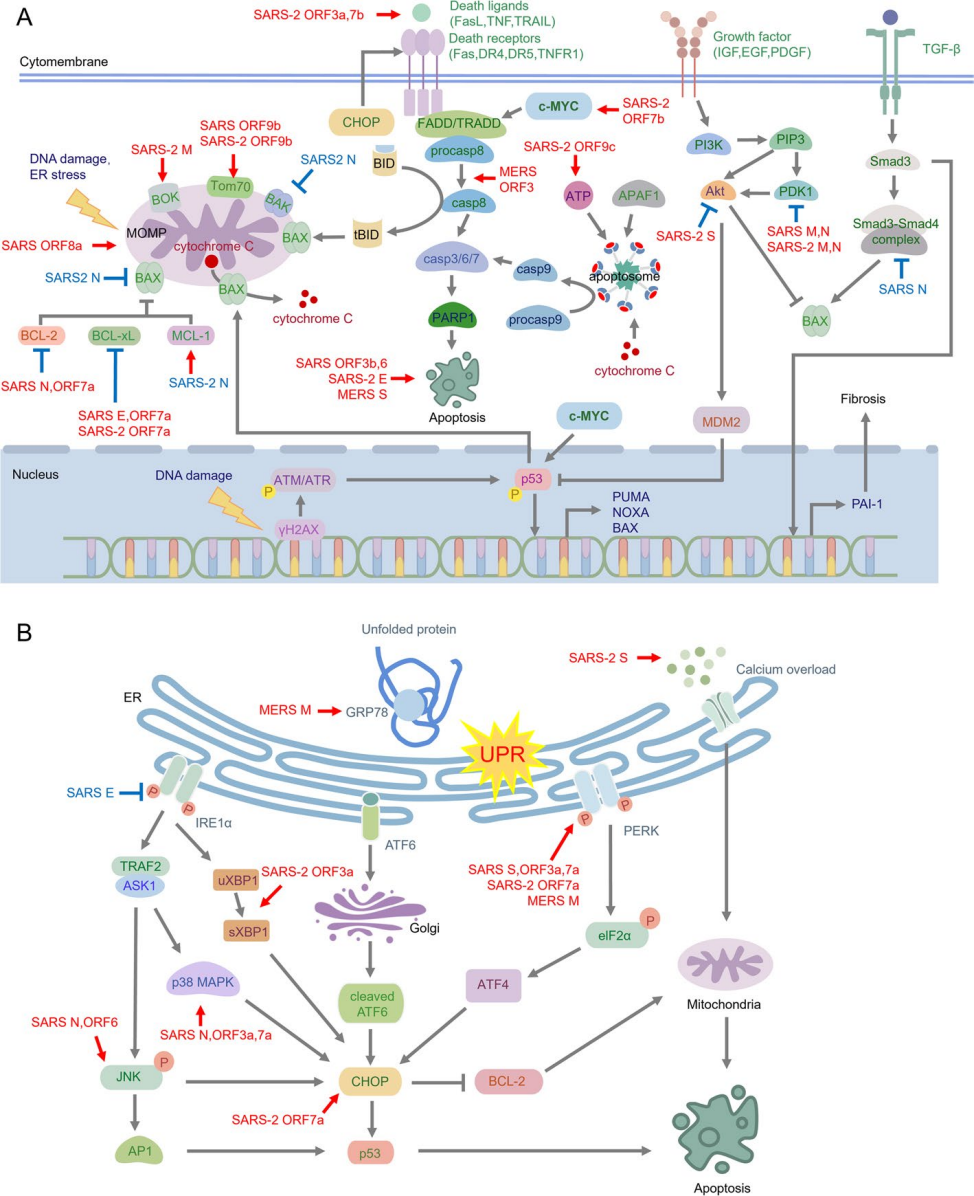
Overview of apoptosis activation by proteins encoded by highly pathogenic coronaviruses. **A**, Exogenous death receptor apoptotic pathway and intrinsic DNA damage-induced apoptosis pathway. **B**, Intrinsic ER stress-induced apoptosis pathway. The majority of proteins encoded by highly pathogenic coronaviruses enhance the activity of pro-apoptotic proteins (indicated by red arrows) and suppress the function of anti-apoptotic proteins (indicated by blue arrows). Certain structural proteins (depicted in blue) exhibit the capability to inhibit apoptosis

Endogenous apoptosis mainly includes ER stress pathway and DNA damage pathway. When DNA damage (such as DNA double-strand break) occurs, DNA damage response (DDR) kinases ataxia-telangiectasia mutated (ATM), ATM- and Rad3-Related (ATR), DNA-dependent protein kinase (DNA-PK) are activated [27], and then a large amount of H2AX is rapidly phosphorylated at Ser-139 to produce phosphorylated histone H2AX (γH2AX) and bind to the damage sites [28], further activating p53 to phosphorylation, and promoting apoptosis by regulating the transcription of apoptosis-related genes (Fig. 3A). When unfolded protein response (UPR) and other factors induce ER stress, the protein kinase RNA–like endoplasmic reticulum kinase (PERK), inositol requiring enzyme 1α (IRE1α), and activating transcription factor 6 (ATF6) pathways are activated, resulting in enhanced C/EBP homologous protein (CHOP) expression and endogenous apoptosis (Fig. 3B). In response to signals such as DNA damage and ER stress, pro-apoptotic BH3-only proteins (BAD, BID, BIM, PUMA, NOXA, etc.) competitively bind to anti-apoptotic proteins (BCL-2, BCL-xL, MCL-1), releasing pro-apoptotic proteins (BAX, BAK, BOK) from anti-apoptotic proteins [29]. Free pro-apoptotic proteins form oligomers, leading to their activation and translocation to the outer mitochondrial membrane, forming channels. These channels cause mitochondrial outer membrane permeabilization (MOMP), resulting in the release of cytochrome C from the intermembrane space of mitochondria into the cytoplasm. Cytochrome C works with procaspase-9 and apoptotic protease activating factor 1 (APAF1) to form apoptosomes, which activates caspase-9. Then, activated caspase-9 cleaves procaspase-3, generating caspase-3. Caspase-3 further cleaves the DNA repair enzyme poly ADP-ribose polymerase (PARP), leading to DNA repair dysregulation and eventually triggering endogenous apoptosis [30–34] (Fig. 3).

The exogenous pathway relies on the activation of death receptors on the cell surface. When extracellular death ligands (such as FasL, TNF and TRAIL) bind to death receptors (Fas, TNFR1, TRAILR1, TRAILR2), a death-inducing signaling complex (DISC) containing the intracellular death domain of the death receptor, Fas-associating death domain protein (FADD)/ TNFR1-associated death domain protein (TRADD) and caspase-8 was formed. Caspase-8 is activated through oligomerization and subsequently, cleaves procaspase-3 to generate caspase-3, eventually exogenous apoptosis. Activated caspase-8, on the other hand, cleaves BH3-interacting domain death agonist (BID) into truncated BID (tBID), promotes the translocation of tBID from the cytosol to the mitochondria, which contribute to MOMP, ultimately leading to apoptosis [30–32, 34] (Fig. 3A).

In conclusion, apoptosis is regulated by complex signal transduction pathways involving both endogenous and exogenous pathways. Understanding the mechanisms and interactions involved in apoptosis signal transduction is crucial for unraveling the intricate processes underlying cell death and survival and developing novel therapeutic strategies targeting apoptosis-related diseases.

### Apoptosis and highly pathogenic coronaviruses: exploring the role of apoptosis in coronavirus pathogenesis

Traditionally, apoptosis has been regarded as a means for host cells to rescue themselves and facilitate viral clearance [35, 36]. However, compelling evidence is emerging to suggest that apoptosis can act as a double-edged sword, capable of benefiting both DNA and RNA viruses in promoting their self-replication [37, 38].

Remarkably, it has been observed that caspase-deficient cells exhibit a heightened antiviral ability compared to normal cells [39]. Mitochondrial stress is one of the ways that SARS-CoV-2 activates the cyclic GMP-AMP synthase-stimulator of interferon gene (cGAS-STING) signaling pathway, which causes mitochondrial dysfunction and releases mitochondrial DNA (mtDNA) from the mitochondria into the cytoplasm [40], resulting in upregulating of type I interferon (IFN-I) expression. In order to antagonize the antiviral effect of interferon, the virus employs a strategy by enhancing the apoptosis signal, activating caspase-3 and caspase-7, both of which play crucial roles downstream in the apoptotic pathway [41]. These activated caspases not only cleave and inactivate IFN-I [42], but also promote mtDNA degradation, and inhibit the activation of cGAS-STING signaling pathway [41, 43]. Consequently, the virus evades the surveillance and clearance by the host immune system, establishing a favorable environment for its survival.

In the long-term struggle between viruses and hosts, some viruses have developed strategies to manipulate cellular processes. For highly pathogenic coronaviruses such as MERS-CoV, SARS-CoV, and SARS-CoV-2 mentioned above, inducing apoptosis is an important way to promote viral replication, aggravate tissue and organ damage, and motivate the development of diseases [37, 38].

Studies have demonstrated that apoptosis is associated with lung injury, multi-organ failure in COVID-19 patients [44–46]. Notably, SARS-CoV-2 predominantly induces apoptosis in respiratory epithelial cells rather than necrosis. This preference is evidenced by the abundance of apoptotic cells and scarcity of necrotic cells following invasion of human respiratory epithelial cells [47]. This characteristic may reflect the virus’s “cleverness” in adopting immune “silencing” apoptosis as a survival strategy given that apoptosis is more suitable for the virus’s survival than necrosis, which can trigger excessive production of inflammatory factors. Following SARS-CoV-2 infection, the virus persists longer in the nasal mucosa compared to the lungs, with lower production of inflammatory factors [48]. This is due to nasal mucosal epithelial cells primarily undergoing apoptosis after viral infection, whereas in the lungs, the apoptosis rate is lower and the pyroptosis rate is higher [48], while apoptosis of immune silence is more conducive to viral replication. Notably, the virus is predominantly present in superficial epithelial cells in the early stages of infection, and gradually invades submucosal cells as the disease progresses [48]. Therefore, apoptosis may favor the spread of the virus from nasal mucosal epithelial cells to submucosal cells, that is, apoptosis can promote the spread of SARS-CoV-2.

Moreover, evidence suggests that SARS-CoV-2 triggers apoptosis in lung epithelial cells, destroys the alveolar capillary barrier, thereby promoting the development of pulmonary edema and acute respiratory distress syndrome (ARDS), and aggravating lung injury in patients with COVID-19, resulting in high mortality [35, 49–52]. Patients with severe COVID-19 were more likely to develop apoptosis than those with mild symptoms, highlighting the direct relationship between apoptosis levels and severity and mortality of COVID-19 patients [53]. During SARS-CoV-2 infection, the apoptosis ratio of B lymphocytes, T lymphocytes [54, 55] and monocytes [53] is also elevated. This coupled with impaired phagocytosis and anti-inflammatory function of macrophages and monocytes after phagocytosing apoptosis cells [56], particularly in severe clinical cases, suggests that enhanced apoptosis of immune cells may contribute to the severe clinical symptoms of COVID-19 patients. Additionally, SARS-CoV-2-induced apoptosis in β pancreatic cells contributes to abnormal glucose metabolism, which aggravates diabetes [57]. In summary, apoptosis aggravates multiple organ failure and microcirculation disorders through various mechanisms, leading to higher patient mortality rates and poor clinical outcomes.

MERS-CoV activates both endogenous and exogenous apoptotic pathways, leading to extensive apoptosis of bronchial epithelial cells, renal cells, macrophages, dendritic cells and other cells, resulting in high morbidity and mortality among MERS patients [58–60]. Due to elevated dipeptidyl peptidase-4 (DPP4) receptor expression on T lymphocytes, they become more vulnerable to MERS-CoV infection, triggering apoptosis. MERS-CoV targets lymphoid organs like the spleen and tonsils, infecting T lymphocytes at various developmental stages, leading to extensive apoptosis and subsequent lymphocyte depletion. This immune system paralysis exacerbates viral infection, culminating in a severe prognosis for patients [59]. Yeung ML et al. discovered that MERS-CoV induces apoptosis in kidney cells by upregulating the expression of smad family member 7 (Smad7) and fibroblast growth factor 2 (FGF2), thus facilitating viral release and dissemination of infection in kidneys and other tissues. Consequently, the incidence of renal failure in MERS patients surpasses that of other human coronavirus infections [58]. Notably, MERS-CoV uses caspase-6, a component of the apoptosis cascade, to cleave the N protein, producing small fragments that act as interferon antagonists and suppress the host immune response, thus promoting replication [38]. The inhibition of caspase-6 can attenuate MERS-CoV replication in human lung tissue and human intestinal organoids, and also improve the pathological changes of the lung in vivo caused by the virus [38].

Furthermore, SARS-CoV infection triggers significant apoptosis in lung epithelial cells [61, 62], lymphocytes [63, 64], liver [65], thyroid [66] and kidney [67] cells. Microarray analysis of host genes showed that the expression of 13 pro-apoptotic genes was up-regulated after SARS-CoV infection, while only 3 pro-apoptotic genes were up-regulated after infection with the low-pathogenic coronavirus HCoV-229E. Thus, highly pathogenic coronaviruses enhance pathogenicity by inducing apoptosis. It is with regret that cross-sectional comparisons of the apoptosis-inducing capacity of highly pathogenic coronaviruses are still lacking in the field. Further study of this may help us understand the important role of apoptosis in the pathogenesis of highly pathogenic coronaviruses.

The induction of apoptosis to promote viral replication is not exclusive to coronaviruses but is a common survival strategy employed by many viruses, including cowpox viruses (CPXV) [68], porcine epidemic diarrhea virus (PEDV) [69, 70], herpes simplex virus (HSV) [71, 72], Epstein-Barr virus (EBV) [73], human immunodeficiency virus (HIV) [74–83], Zika virus (ZIKV) [84], Hepatitis C virus (HCV) [85–92], and others. Therefore, the virus exploits apoptosis to enhance its replication, leading to a substantial increase in the number of infected cells undergoing apoptosis, aggravating the patient’s condition. Apoptosis serves as a crucial tactic for viruses to suppress host immune responses and facilitate infection. Understanding the intricate interplay between apoptosis and viral infectious diseases is vital for deciphering the complexities underlying viral pathogenesis. Further exploration of these mechanisms holds promise for the development of innovative strategies to combat apoptosis-related viral diseases.

### Mechanism and biological significance of apoptosis modulated by highly pathogenic coronavirus structural proteins

Highly pathogenic coronavirus structural proteins, including S, E, M and N proteins, play critical roles in cell invasion, virus particle synthesis, release, and also modulation of cell apoptosis [68–70, 93] (Table 1).

**Table 1:** Summary of apoptosis modulated by structural proteins of highly pathogenic coronaviruses

Coronavirus	Structural protein	Effect on apoptosis	Molecular mechanism	Biological significance	Reference
SARS-CoV-2	S	Promote	1.Inhibit PI3K/Akt/mTOR pathway 2.Disrupt intracellular calcium homeostasis		[71, 72]
	E	Promote			[77]
	M	Promote	1.Inhibit BOK ubiquitination 2.Inhibit PDK1-PKB/Akt pathway	Damage to the alveolar capillary barrier	[52, 80–83]
	N	Suppress	Stabilize MCL-1	Promote viral replication without causing respiratory dysfunction and increases the risk of superinfection	[87, 88]
SARS-CoV	S	Promote	Activate the PERK pathway	Promote viral replication	[73–75]
	E	Promote	1.Trigger ER stress 2.Prevent BCL-xL translocation to mitochondria		[63, 78]
		Suppress	Inhibit IRE1α pathway		[79]
	M	Promote	Inhibit PDK1-PKB/Akt pathway		[82, 84]
	N	Promote	Up-regulated JNK and p38 MAPK pathway, and down-regulated Akt phosphorylation and BCL-2 protein level		[24, 85]
		Suppress	Interfere with Smad3-Smad4 complex formation	Increase apoptosis and exacerbate tissue fibrosis	[86]
MERS-CoV	S	Promote			[76]
	M	Promote	Disrupt the binding of GRP78 and PERK, thereby specifically activating the PERK pathway	Promote apoptosis, enhance viral replication, and aggravate lung injury	[37]

Specifically, the SARS-CoV-2 S protein induces apoptosis through various pathways. Firstly, SARS-CoV-2 S promotes apoptosis through autophagy by reactive oxygen species (ROS)-suppressed PI3K/AKT/mTOR signaling [71]. Moreover, S protein directly interacts with the major receptor angiotensin-converting enzyme 2 (ACE2), facilitating the formation of ACE2-calcium channel clusters. This interaction causes overactivation of calcium channels, disrupting intracellular calcium homeostasis, and ultimately inducing apoptosis [72] (Fig. 3). Furthermore, SARS-CoV S protein triggers ER stress and UPR through PERK pathway activation, resulting in disrupted cellular homeostasis, apoptosis, and enhanced viral replication [73–75] (Fig. 3B). Additionally, the induction of apoptosis by MERS-CoV S protein has also been reported, although the specific regulatory mechanism remains unclear [76] (Fig. 3A).

SARS-CoV-2 E protein has been shown to induce apoptosis in periodontal ligament fibroblasts [77], although the mechanism of action remains to be elucidated (Fig. 3A). Studies have reported that SARS-CoV E proteins can trigger ER stress [63, 78] and promote mitochondria-mediated apoptosis by sequestering the anti-apoptotic protein B-cell lymphoma-extra large (BCL-xL) onto the ER membrane [63] (Fig. 3A). Remarkably, SARS-CoV E protein has been reported to hinder the process of apoptosis. For example, they can down-regulate the IRE1α signaling pathway associated with unfolded proteins, without affecting the PERK and ATF6 signaling pathways (Fig. 3B). Ultimately, this reduction in apoptosis occurs [79]. The fact that the same viral protein has opposite effects on apoptosis may indicate that the viral protein performs different functions at different stages of the viral life cycle.

SARS-CoV-2 M protein induces apoptosis in lung epithelial cells through mitochondria, and can inhibit BOK ubiquitination and stabilize its levels by interacting with the BH2 domain of BOK’s endodomain in the absence of BAX and BAK, ultimately promoting BOK mitochondrial localization [52] (Fig. 3A). BOK can directly mediate the increase of mitochondrial membrane permeability and ultimately activate mitochondrial pathway-induced apoptosis [80]. In addition, SARS-CoV-2 M protein can also inhibit the activation of PDK1-Akt signaling and induce caspase-dependent apoptosis by interacting with 3-phosphoinositide-dependent protein kinase 1 (PDK1), while the SARS-CoV-2 N protein can specifically enhance the interaction between M protein and PDK1, thereby enhancing apoptosis induced by M protein [81–83] (Fig. 3A). The synergistic effect of M proteins and N proteins to enhance their respective levels of induced apoptosis has also been found in SARS-CoV [82, 84] (Fig. 3A). Meanwhile, the MERS-CoV M protein can interact with the ER marker glucose regulated protein 78kD (GRP78), disrupt the binding of GRP78 and PERK, specifically activate the PERK pathway, further activate the expression of downstream pro-apoptotic genes (Fig. 3B), enhance self-replication, aggravate host lung injury, and increase the susceptibility to apoptosis inducer, Etoposide. In addition, PERK inhibitor significantly inhibits MERS-CoV replication [37].

SARS-CoV N protein activates apoptosis signaling by up-regulating Jun-N-terminal kinase (JNK) and p38 mitogen-activated protein kinase (p38 MAPK) pathways, while down-regulating Akt phosphorylation and BCL-2 levels [85] (Fig. 3). Conversely, the N protein also demonstrates anti-apoptotic capabilities. SARS-CoV N protein specifically binds to Smad3 through the MH2 domain, interfering with the formation of the Smad3-Smad4 complex. This results in increased transcription of transforming growth factor-β (TGF-β), promoting plasminogen activator inhibitor-1 (PAI-1) expression and aggravating tissue fibrosis post-infection. However, it weakens the Smad3-Smad4 complex-mediated apoptosis [86] (Fig. 3A). Among the proteins encoded by SARS-CoV-2, the N protein has the function of inhibiting apoptosis by regulating apoptosis-related genes (BAX, BAK, BCL-2) [87]. The PAN P’s team [88] discovered that the SARS-CoV-2 N protein specifically interacts with the anti-apoptotic protein myeloid cell leukemia-1 (MCL-1), recruiting the deubiquitinating enzyme ubiquitin-specific peptidase 15 (USP15) to remove K63 ubiquitination of MCL-1 and stabilize MCL-1 to inhibit BAK’s function in mitochondria, and ultimately inhibiting apoptosis (Fig. 3A). N protein promotes viral replication, such as influenza A virus (IAV), dengue virus (DENV), and ZIKV, exacerbating mortality in infected mice. All of these can be blocked by MCL-1-specific inhibitors [88]. SARS-CoV-2 replicates effectively in asymptomatic patients without causing respiratory dysfunction. However, it increases the risk of superinfection, possibly attributed to inhibition of apoptosis by N proteins [88].

The same viral protein can result in different effects on apoptosis, which may be related to the different effects of apoptosis on different stages of the viral life cycle. Understanding these mechanisms and the significance of coronavirus structural proteins-induced apoptosis is crucial in developing therapeutics and interventions against highly pathogenic coronaviruses.

### Mechanism and biological significance of apoptosis induced by highly pathogenic coronaviruses non-structural proteins

The proteins (NSP1-NSP16) encoded by the ORF1ab of highly pathogenic coronavirus are mainly involved in the transcriptional replication [89], with limited reports on their apoptosis-inducing abilities. However, certain non-structural proteins, such as ORF3a [16], ORF6 [19, 21–24, 90], ORF7a [20, 91], ORF7b [21, 91], and ORF8 [25, 92, 94] possess functions of antagonizing innate immune signaling and assisting viral invasion of host immune responses. Moreover, these non-structural proteins also play a crucial role in inducing apoptosis (Table 2).

**Table 2:** Summary of apoptosis induced by non-structural proteins of highly pathogenic coronaviruses

Coronavirus	Non-structural protein	Effect on apoptosis	Molecular mechanism	Biological significance	Reference
SARS-CoV-2	ORF3a	Promote	1.Upregulate death receptors and ligands 2.Activation of ER stress via reticulophagy		[36, 95, 96]
	ORF7a	Promote	1.C-terminus interacts with BCL-xL and recruits BCL-xL to ER to activate ER stress 2.Up-regulate CHOP and activate ER stress		[97]
	ORF7b	Promote	Up-regulate c-MYC and thus promote TNFα expression	Mediate apoptosis to cause lung damage	[98, 99]
	ORF9b	Promote	Binding to Tom70, apoptosis is mediated by Tom70/Hsp90/IRF3/Bax complex	Inhibit IFN-I signaling through Tom70	[100–105]
	ORF9c	Promote	Affect ATP metabolism and induce transcription levels of pro-apoptotic genes	Cause cardiomyocyte apoptosis, leading to COVID-19 related heart damage	[106]
SARS-CoV	ORF3a	Promote	Activation of MAPK or PERK pathways regulates ER stress	Facilitate the packaging and release of viruses	[62, 95, 107–110]
	ORF3b	Promote	Induce G0/G1 arrest and apoptosis		[111, 112]
	ORF6	Promote	Mediate JNK-dependent ER stress		[113]
	ORF7a	Promote	1.Pro-apoptotic mechanism is similar to SARS-CoV-2 ORF7a 2.Activate the MAPK pathway, inhibit cell translation and induce apoptosis		[114, 115]
	ORF8a	Promote	Affect mitochondrial potential	Promote viral infection	[116–118]
	ORF9b	Promote	1.Binding to Tom70, apoptosis is mediated by Tom70/Hsp90/IRF3/Bax complex 2.ORF9b retained in the nucleus regulates apoptosis-related transcription factors	Inhibit IFN-I signaling through Tom70	[100–105, 119]
MERS-CoV	ORF3	Promote	Activate death receptors		[120]

SARS-CoV ORF3a and SARS-CoV-2 ORF3a use different strategies to induce apoptosis [36]. SARS-CoV-2 ORF3a, localized in the ER, initiates RETREG1/FAM134B-associated ER autophagy (Reticulophagy) [116], activating IRE1α-mediated spliced X-box-protein 1 (sXBP1) production, thereby inducing apoptosis via the endogenous ER stress pathway [117] (Fig. 3B). In addition, SARS-CoV-2 ORF3a upregulates death receptor ligands and activates apoptosis induced by exogenous death receptor pathway [36] (Fig. 3A). On the other hand, SARS-CoV ORF3a activates the p38 MAPK pathway or activates the PERK pathway to regulate apoptosis induced by ER stress pathway [62, 95, 107, 108], affecting the packaging and release of the virus [109, 110], and PERK kinase inhibitors can significantly reduce apoptosis and inflammation in lung epithelial cells [62] (Fig. 3B).

ORF3 of MERS-CoV has similar ability to induce apoptosis as SARS-CoV ORF3a and SARS-CoV-2 ORF3a, mediating apoptosis through the exogenous death receptor pathway in a dose-dependent manner (Fig. 3A). However, ORF3 is less stable than SARS-CoV ORF3a and SARS-CoV-2 ORF3a as it is easily ubiquitinated and degraded by the host E3 ligase HUWE1, leading to reduced apoptosis induction potential and possibly contributing to its lower transmissibility [120].

SARS-CoV ORF3b can induce G0/G1 arrest and apoptosis [111, 112] (Fig. 3A), while the function of SARS-CoV-2 ORF3d, which was previously mistaken for SARS-CoV-2 ORF3b, remains unknown. SARS-CoV ORF6 is known to mediate ER stress and JNK-dependent apoptosis [113] (Fig. 3B). However, further research is needed to determine whether SARS-CoV-2 ORF6 can induce apoptosis. It should be noted that MERS-CoV ORF6 acts as the E protein and will not be discussed further in this context.

SARS-CoV-2 ORF7a interacts with the anti-apoptotic protein BCL-xL through the C-terminal amino acid residues Lys117 and Lys 119, recruiting BCL-xL to the ER to activate ER stress and induce apoptosis (Fig. 3A). Concurrently, ORF7a induces ER stress through the PERK-elF2α-CHOP pathway, suppressing the expression of endogenous BCL-xL and thereby augmenting apoptosis (Fig. 3B). The ubiquitination of ORF7a Lys119 can diminish its interaction with BCL-xL, impeding the aggregation of BCL-xL in the ER, and consequently preventing ER stress and inhibiting apoptosis [97]. Remarkably, SARS-CoV ORF7a exhibits a similar pro-apoptotic mechanism to SARS-CoV-2 ORF7a [114]. Moreover, SARS-CoV ORF7a can also activate the p38 MAPK pathway, inhibit the host cell translation process and induce apoptosis [115] (Fig. 3B).

It has been reported that SARS-CoV-2 ORF7b upregulates MYC proto-oncogene (c-MYC) and induces c-MYC signaling to promote death receptor-mediated apoptosis and aggravate tissue and organ damage [98, 99] (Fig. 3A). Remarkably, SARS-CoV ORF7b localizes to the Golgi [121] and serves not only as a non-structural protein, but also participates in the assembly of SARS-CoV viral particles with ORF3a, ORF7a and ORF9b [7, 8] (Fig. 2A). However, the induction of apoptosis by SARS-CoV ORF7b has yet to be established.

ORF8 emerges as a notable viral protein, particularly noteworthy due to the observed deletion in 90% of SARS-CoV-2 strains [122–126]. This deletion is implicated in enhancing the virus’s adaptability and facilitating its global dissemination [124], often correlating with milder manifestations of COVID-19 [122]. SARS-CoV-2 ORF8 shares a striking 95% homology with Bat-CoV (RaTG13) ORF8, while exhibiting only 30% amino acid sequence similarity with SARS-CoV ORF8ab [127]. The latter originates predominantly from greater horseshoe bats, *Rhinolophus ferrumequinum* (SARSr-Rf-BatCoV) and Chinese horseshoe bats, *Rhinolophus sinicus* (SARSr-Rs-BatCoV) [128, 129]. During early propagation, a 29-nucleotide deletion led to the splitting of the original ORF8 into ORF8a and ORF8b [130, 131]. Previous investigations suggest that neither SARS-CoV-2 ORF8 nor ORF10 possess apoptotic-inducing capabilities [132]. Interestingly, SARS-CoV ORF8a promote apoptosis and facilitates viral infection through a mitochondria-dependent pathway [116, 117], possibly attributed to its mitochondrial localization [116, 118] (Fig. 3A). On the other hand, SARS-CoV ORF8b induces cell death through a distinct mechanism, accumulating intracellularly and leading to ER stress and autophagy. This cascade triggers the activation of NOD-, LRR- and pyrin domain-containing protein 3 (NLRP3) inflammasomes within lung epithelial cells, and ultimately in pyroptosis [133].

Both SARS-CoV-2 ORF9b and SARS-CoV ORF9b localize to the mitochondria and interact with the translocase of outer mitochondrial membrane 70 (Tom70) to form the Tom70/Hsp90/IRF3/Bax complex, which inhibits IFN-I signaling and promotes apoptosis [100–105] (Fig. 3A). Meanwhile, the apoptosis is significantly increased when the nuclear export of ORF9b is blocked, which may be related to the interaction of ORF9b with apoptosis-related transcription factors in the nucleus [101].

Some studies suggest that SARS-CoV-2 ORF9c may enhance the transcription of apoptosis-related genes in human cardiomyocytes by influencing adenosine 5’-triphosphate (ATP) metabolism, which may lead to heart disease induced by COVID-19 [106] (Fig. 3A). It has been observed that the administration of Ivermectin and Meclozine can restore cellular ATP levels and ameliorate SARS-CoV-2 ORF9c-induced cardiomyocyte apoptosis and dysfunction [106].

In summary, although some viral proteins are still unknown whether they can regulate apoptosis, existing reports have shown that non-structural proteins encoded by highly pathogenic coronaviruses induce apoptosis and promote viral survival through multiple mechanisms. In vitro experiments have demonstrated that apoptosis inhibitors can significantly inhibit the replication of highly pathogenic coronaviruses [37]. This further underscores the critical role of apoptosis in the survival of these virus.

### The important role of expressing more non-structural proteins by highly pathogenic coronaviruses in virus-induced cell apoptosis

Highly pathogenic coronaviruses exhibit heightened replication efficiency and transmissibility compared to their low-pathogenic counterparts [47], potentially due to the increased abundance of non-structural proteins they encode (Fig. 2B). While research on the induction of apoptosis by low-pathogenic coronaviruses is limited [134–136], existing studies suggest that highly pathogenic coronaviruses promote the expression of more pro-apoptotic genes [134–136]. Non-structural proteins by highly pathogenic coronaviruses plays a pivotal role in inducing cell apoptosis through multiple mechanisms. Importantly, innate immunity is also impaired during the process, which benefits the virus's replication and dissemination within the host, leading to exacerbated pathology and disease severity (Table 2). Understanding this mechanism is essential for comprehending the virulence and pathogenesis of highly pathogenic coronaviruses, offering insights for the development of targeted therapeutic interventions aimed at mitigating virus-induced cell apoptosis and reducing disease severity. Ultimately, unraveling the intricacies of non-structural protein-mediated apoptosis sheds light on potential avenues for effective intervention against highly pathogenic coronaviruses and related viral diseases.

### Application and limitations of apoptosis inhibitors in antiviral research

The use of apoptosis inhibitors has shown promise in inhibiting coronavirus-induced apoptosis and attenuating viral replication. To explore the potential of apoptosis inhibitors as antiviral drugs, with a focus on their application in the field of coronaviruses is meaningful. Additionally, the limitations and the need for further research and improvement in the clinical application of apoptosis inhibitors against viruses should be discussed.

Highly pathogenic coronaviruses use caspase family members to promote viral survival and immune evasion. In vitro and in vivo experiments have proved that caspase members have the function of antagonizing key proteins of innate immune pathway and helping viral immune evasion, and caspase inhibitors effectively inhibit the replication of coronavirus, alleviate lung damage and excessive immune response in mice caused by viral infection, and significantly improve the survival rate [37, 38]. It is worth noting that caspase-6 inhibitors did not affect the replication of influenza virus (H1N1) or enterovirus (EV-A71), and it is possible that apoptosis has a significant effect on the replication of coronavirus, but not on other viruses [38]. Despite this, different inhibitors of the apoptotic pathway exhibit varying efficacy against different coronaviruses. For example, caspase-6 and PERK inhibitors have a significant therapeutic effect on MERS-CoV-infected mice but have poor efficacy on SARS-CoV and SARS-CoV-2 infected mice [37, 38]. Understanding the specific caspase proteins involved in viral replication and identifying corresponding inhibitors are crucial for the treatment of highly pathogenic coronaviruses.

In current coronavirus research, inhibiting apoptosis activation through apoptosis inhibitors has shown promise in curbing viral replication. Certain PARP inhibitors, including Olaparib [137–139], Stenoparib [140], and CVL218 [141, 142], exhibit antiviral effects against highly pathogenic coronaviruses.They effectively control viral replication, mitigate inflammatory responses, and alleviate pathological changes. Combining PARP inhibitors with Remdesivir enhances their efficacy in inhibiting coronaviruses, presenting a significant potential in treating highly pathogenic coronavirus infections. While some apoptosis inhibitors have demonstrated efficacy in animal and cell studies, their clinical application as antiviral drugs lacks established protocols and requires further refinement and research.Furthermore, the susceptibility of other mammals to coronavirus infection and their reliance on caspase remains unclear. Considering the reports that swine acute diarrhea syndrome coronavirus (SADS-CoV) may spread across species and become the next zoonotic coronavirus [143], the study on investigating the role of caspase in mammalian susceptible coronaviruses could aid in timely response to outbreaks and facilitate the development of effective therapeutic drugs.

However, the clinical application of apoptosis inhibitors for the treatment of viral infections is currently limited due to associated side effects. Studies have indicated that certain caspase proteins inhibit the production of IFN-I, a key mediator of antiviral immune responses. It has been shown that caspase-9, caspase-3, and caspase-7 can inhibit the production of IFN-I mediated by the cGAS-STING signaling pathway [41, 43]. In human cells, caspase-3 suppresses IFN-I in a manner independent of mtDNA, reduces cytokine release by cleaving cGAS, mitochondrial antiviral signaling protein (MAVS), and interferon regulatory factor 3 (IRF3) [39], and silences apoptotic cell immunity, as is caspase-7 in mice cells [39]. As a consequence, the suppression of caspases’s function may probably lead to excessive immune activation and inflammatory cytokine storm, posing a risk during the treatment of patients with coronavirus infection. Therefore, addressing the issue of potential side effects is crucial in the development of apoptosis inhibitors for antiviral therapy.

## Conclusion remarks

Apoptosis plays an important role in both physiological processes and the pathogenesis of highly pathogenic coronaviruses, as well as an important driver of disease progression. More and more studies have proved that apoptosis serves not only as a means for the host to reduce viral replication and facilitate viral clearance, but also as a strategy employed by viruses to antagonize the host immune system surveillance and response, as well as exploit apoptosis and its components to suppress the production of antiviral factors, bolster viral replication, and augment infectivity.

While SARS-CoV-2 infection has been linked to various modes of cell death, including apoptosis, pyroptosis, and ferroptosis [45, 48, 144, 145], studies indicate that the lungs have a higher proportion of pyroptosis and necroptosis, while the upper respiratory tract, with a greater viral load, tends towards apoptosis. [48, 146]. This observatiobsuggests that apoptosis imay be more conducive to viral replication than pyroptosis and necroptosis. Moreover, the latest in vivo experimental evidence suggests that necroptosis has no significant effect on viral transmission, disease pathology, or early host immune responses, independent of disease progression [147]. Consequently, targeting mixed lineage kinase domain-like pseudokinase (MLKL), a key protein in the necroptotic pathway, with antiviral drugs may yield limited effectiveness.High pathogenic strains have evolved additional non-structural proteins that regulate apoptosis in tandem with the encoded structural proteins. Simultaneously, these non-structural proteins promote viral survival in a way that antagonize innate immunity. Although not all virus-encoded proteins induce apoptosis, many reported apoptosis-related proteins indeed trigger this process, and each protein is presumed to play a role in viral replication regulation. This phenomenon likely stems from the necessity, during the early stages of virus invasion, to maintain host cell integrity to facilitate optimal conditions for viral replication. Structural proteins introduced into cells during viral invasion play a role in inhibiting apoptosis at this stage. However, upon completing replication, the virus aims to release a multitude of viral particles from the cell through apoptosis, thereby dampening the host immune response and diminishing the likelihood of elimination. Consequently, numerous virus-encoded proteins are involved in regulating apoptosis induction. Different viral proteins possess varying abilities to induce apoptosis at distinct stages of viral replication, targeting diverse pathways to ensure apoptosis of virus-infected cells. Highly pathogenic coronaviruses rely on a larger array of non-structural proteins to modulate apoptosis, a process pivotal in augmenting viral replication, exacerbating tissue and organ damage, and advancing disease progression. In summary, apoptosis contributes to multiple organ failure and microcirculation disorders through diverse mechanisms, resulting in elevated patient mortality rates and unfavorable clinical outcomes.The high pathogenicity of coronavirus-encoded proteins in efficiently inducing apoptosis has garnered interest in their potential use for treating small cell lung cancer. Specifically, the S proteins of SARS-CoV-2 have been shown to induce apoptosis and can successfully induce tumor cell apoptosis in mice models when administered intranasally [148]. However, it is regrettable that this has only been verified in animal experiments, and further research is necessary to bolster the validation of clinical trials and eventually apply them to clinical practice. It has also been reported that in vitro experiments, lentiviral particles Gag-CASP8-VLPs, carrying activated caspase-8 and constructed using the VSV G protein (VSV-G) as the vector, can enter breast cancer cells and inhibit tumor cell growth [149]. In the future, small molecule drugs that mimic the structure of the key functional domains of highly pathogenic coronavirus proteins may be applied to target tumor cells and induce tumor cell death, providing a new perspective in tumor treatment.

Here, we systematically summarized the mechanism and biological significance of apoptosis induced by highly pathogenic coronaviruses structural and non-structural proteins, caspase-mediated survival strategies in highly pathogenic coronaviruses and the potential of apoptosis inhibitors in antiviral research. Further study should focus on the investigating the network-based research on viral proteins and key compositions in the apoptosis pathway. Furthermore, combining this knowledge with other antiviral medicines may help inhibit viral infection and alleviate tissue and organ damage. By simulating how highly pathogenic coronavirus-encoded proteins activate apoptosis, researchers can identify or design small molecule drugs with the ability to target and activate apoptosis in tumor cells. These efforts could unlock new insights into the biological function of highly pathogenic coronavirus proteins and the regulatory mechanism of apoptosis. Such in-depth research holds great significance in our understanding of the pathogenic mechanisms of coronaviruses, the development of effective treatment strategies, and the prevention of new infectious diseases.

## Data Availability

All data relevant to this review are included in the text, references, tables and figures.

## Abbreviations

ACE2: Angiotensin-converting enzyme 2
AKT: Protein kinase B
AP1: Activator protein 1
APAF1: Apoptotic promoting factor 1
ARDS: Acute respiratory distress syndrome
ASK1: Apoptotic-signaling kinase 1
ATF4: Activating transcription factor 4
ATM: Ataxia-telangiectasia mutated
ATP: Adenosine 5'-triphosphate
ATR: ATM- and Rad3-related
BAD: BCL2 associated agonist of cell death gene
BAK: BCL2 antagonist/killer 1
BAX: BCL2-associated protein X
BCL-2: B-cell lymphoma-2
BCL-xL: B-cell lymphoma-extra large
BID: BH3-interacting domain death agonist
BIM: BCL-2 interacting mediator of cell death
BOK: BCL-2-related ovarian killer
Caspase: Cysteinyl aspartate specific proteinase
cGAS: Cyclic GMP-AMP synthase
CHOP: C/EBP homologous protein
c-MYC: MYC proto-oncogene
COVID-19: Corona Virus Disease 2019
CPXV: Cowpox viruses
DDR: DNA damage response
DENV: Dengue virus
DISC: Death-inducing signaling complex
DNA-PK: DNA-dependent protein kinase
DPP4: Dipeptidyl peptidase 4
E: Envelope
EBV: Epstein-barr virus
EGF: Epidermal growth factor
elF2α: Eukaryotic initiation factor 2α
ER: Endoplasmic reticulum
FADD: Fas-associating death domain protein
Fas: Factor-related apoptosis
FasL: Factor-related apoptosis ligand
FGF2: Fibroblast growth factor 2
GRP78: Glucose regulated protein 78kD
HCoV: Human coronavirus
HCV: Hepatitis C virus
HIV: Human immunodeficiency virus
HSP90: Heat shock protein 90
HSV: Herpes simplex virus
IAV: Influenza A virus
IFN-I: Type I interferon
IGF: Insulin like growth factor
IRE1α: Inositol requiring enzyme 1α
IRF3: Interferon regulatory factor 3
JNK: Jun-N-terminal kinase
M: Membrane
MAVS: Mitochondrial antiviral signaling protein
MCL-1: Myeloid cell leukemia-1
MDM2: Murine double minute 2
MERS-CoV: Middle east respiratory syndrome coronavirus
MLKL: Mixed lineage kinase domain-like pseudokinase
MOMP: Mitochondrial outer membrane permeabilization
mtDNA: Mitochondrial DNA
mTOR: Mammalian target of rapamycin
N: Nucleocapsid
NLRP3: NOD-, LRR- and pyrin domain-containing protein 3
NOXA: Phorbol-12-myristate-13-acetate-induced protein 1
p38 MAPK: P38 mitogen-activated protein kinase
PAI-1: Plasminogen activator inhibitor-1
PARP1: Poly ADP-ribose polymerase 1
PDGF: Platelet derived growth factor
PDK1: 3-Phosphoinositide-dependent protein kinase 1
PEDV: Porcine epidemic diarrhea virus
PERK: Protein kinase RNA–like endoplasmic reticulum kinase
PI3K: Phosphoinositide 3-kinase
PIP3: Phosphatidyl-inositol,3,4,5-triphosphate
PUMA: P53 upregulated modulator of apoptosis
Reticulophagy: ER autophagy
RETREG1/FAM134B: Reticulophagy regulator 1
ROS: Reactive oxygen species
S: Spike
SADS-CoV: Swine acute diarrhea syndrome coronavirus
SARS-CoV-2: Severe acute respiratory syndrome coronavirus 2
Smad: Smad family member
STING: Stimulator of interferon gene
sXBP1: Spliced X-box-protein 1
TGF-β: Transforming growth factor-β
TNF: Tumor necrosis factor
TNFR1: Tumor necrosis factor receptor 1
Tom70: Translocase of outer membrane 70
TRADD: TNFR1-associated death domain protein
TRAF2: TNF receptor associated factor 2
TRAIL: TNF-related apoptosis-inducing ligand
TRAILR1: Tumor necrosis factor receptor 1
TRAILR2: Tumor necrosis factor receptor 2
UPR: Unfolded protein response
USP15: Ubiquitin-specific peptidase 15
ZIKV: Zika virus
γH2AX: Phosphorylated histone H2AX
